# Fatigue Crack Growth in Metallic Materials

**DOI:** 10.3390/ma16010011

**Published:** 2022-12-20

**Authors:** Fernando Ventura Antunes

**Affiliations:** Centre for Mechanical Engineering, Materials and Processes (CEMMPRE), Department of Mechanical Engineering, University of Coimbra, 3030-788 Coimbra, Portugal; fernando.ventura@dem.uc.pt

## 1. Introduction

Mechanical components and structures are submitted to cyclic loads in different applications; therefore, they must be designed to withstand fatigue. In the damage tolerance approach, initial cracks are assumed to exist inside the specimen and to propagate, ultimately leading to failure. The definition of the time between inspections or the time needed to replace the component requires an accurate knowledge of fatigue crack growth (FCG) rate.

Despite significant research advances in recent decades, further studies are needed to accurately model FCG and understand its fundamental mechanisms. This phenomenon is complex and diverse, involving different damage mechanisms, such as cyclic plastic deformation, growth and coalescence of microvoids, environmental damage and brittle failure. Crack tip shielding mechanisms, such as crack branching or crack closure, may also affect FCG rate (da/dN). These competing mechanisms greatly depend on material, load parameters, geometry, environment and temperature. Different parameters have been used as crack driving forces: ΔK, K_max_, J-integral or CTOD. The appearance of new metallic alloys, and the development of new technologies, such as additive manufacturing, introduces additional challenging complexities.

The developments in numerical simulations and experimental research provide insights into this phenomenon. This Special Issue aims to focus on the recent advances in this attractive field of research, which requires a multidisciplinary approach. This issue contains 19 highly diverse papers from leading scientists across the world, especially those with expertise in fatigue failure. A brief overview of these papers is provided below to highlight the multidisciplinary nature and quality of this research. The papers are organized in three main topics: (i) fundamental studies, (ii) applications, and (iii) the development of new tools.

## 2. Fundamental Studies

### 2.1. Online Discussion on Recent Advances in FCG

Daniel Kujawski [1] presented an interesting discussion about FCG issues involving well-known researchers. Due to COVID-19 spreading worldwide in the summer of 2020, many universities were in lockdown and international conferences were cancelled. Therefore, Kujawski organized online meetings. The main theme of these meetings was the *recent advances in FCG investigations and modelling*. The study of [1] presents one of these energetic online discussions, which took place just before meeting #8, held on 26 September 2020. The comments were assembled in the order that they were posted online. The initial question was the effect of changing the minimum load on the crack opening level, while maintaining the maximum load. This discussion was very lively, albeit demonstrating that there is no consensus about the best approach to explain and quantify fatigue phenomenon. Two main lines of thinking emerged from this discussion: (1) those who believe that crack closure is fundamental to explaining different issues of FCG; (2) those who believe that da/dN depends on K_max_ and ΔK, while crack closure phenomenon has no relevant effect.

### 2.2. Crack Driving Force: Non-Linear Parameters

The relevance of crack closure was pointed out in the research of Borges et al. [2], Neto et al. [3] and Sérgio et al. [4]. A numerical approach was adopted by Borges et al. [2] to predict FCG rate, assuming that cyclic plastic deformation is the major cause of damage and using a cumulative plastic strain as the crack driving parameter. Crack growth was simulated by node release when the cumulative plastic strain reached a critical value, calibrated using only one experimental value of da/dN. ∆K was found to control crack tip damage, while K_max_ had no effect. The analysis of FCG after an overload with and without contact of crack flanks showed that the typical variation of da/dN observed is linked to crack closure variations. However, the analysis of crack tip plastic deformation also shows that there is crack tip damage below closure; therefore, the definition of an effective load range ∆K_eff_ = K_max_ − K_open_ is not correct. 

Numerical models are very interesting for developing parametric studies and identifying the mechanisms behind the observed variations. Therefore, the same numerical approach was used by Neto et al. [3] to study the effect of residual stresses on FCG. Artificial thermal residual stresses (TRS) were produced by heating a region ahead of the crack tip. The crack propagation in the compressive residual stress field caused a decrease in the FCG rate, while the regions with compressive TRS showed a decrease in da/dN. A perfect match exists between the trends of da/dN and the original profile of TRS. The analysis of situations without contact of crack flanks increased the FCG rate and, more importantly, eliminated the influence of TRS. This clearly indicates that the TRS affect da/dN indirectly, through plasticity-induced crack closure (PICC).

**Figure 1:**
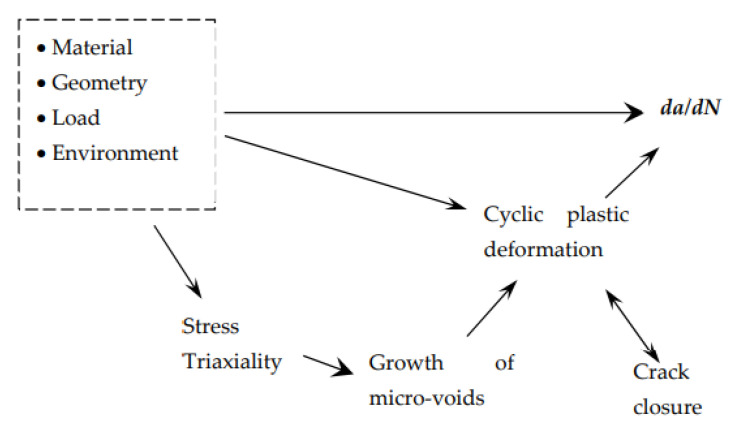
Schematic illustration of mechanisms and parameters involved in FCG modelling [4].

In the same line of research, Sérgio et al. [4] included the effect of the growth of microvoids in the numerical model based on cumulative plastic strain, using the Gurson–Tvergaard–Needleman (GTN) damage model. The main conclusions are: (i) The inclusion of micro-voids produced an unexpected decrease in da/dN for low values of ∆K, while at relatively high values of ∆K, the GTN model increased da/dN. (ii) The inclusion of porosity increases the plastic deformation as well as the size of the plastic zones ahead of the crack tip. (iii) At low values of ∆K, the inclusion of micro-voids increased PICC, reducing da/dN. At high values of ∆K, there was no PICC even with GTN. Disabling the contact of crack flanks caused an increase in da/dN with GTN for all studied values of ∆K. (iv) There is a global trend of an increase in porosity with plastic strain. However, oscillatory behaviour is observed in each load cycle since the stress at the crack tip is of a compressive nature during the unloading phase. The increase in crack length, and thus ∆K, increased the porosity level. (v) There is a strong link between stress triaxiality and porosity level, as illustrated in Figure 1.

The use of non-linear parameters, such as the cumulative plastic strain [1,2,3] represents an evolution in the modelling of FCG. The evolution of numerical methods and experimental techniques enabled a closer look at the crack tip. Different non-linear parameters can be used, such as the plastic CTOD or the size of the crack tip plastic zone, but well-defined relations exist between all parameters. The size of plastic zone at the crack was measured in the study by Lopez-Crespo et al. [5] using Vickers micro-indentation. The volume of material within the plastic zone was plastically deformed, and thus hardened. The experiment and sensitivity analysis showed that polishing the surface to a #3 µm finish and applying a 25 g-force load for 15 s produced the best results in terms of the resolution and quality of the data. The methodology allowed the size and shape of both the cyclic and the monotonic plastic zones to be visualised through 2D contour maps.

### 2.3. Effect of Material Microstructure

FCG greatly depends on the material and its microstructure. Jambor et al. [6] studied 304 L austenitic stainless steel in two microstructural states: as-received (AR) with a finer microstructure and low susceptibility to the transformation-induced plasticity (TRIP) effect, and solution-annealed (SA) with coarser microstructure and higher susceptibility to TRIP. The tests at R = 0.1 revealed a higher threshold value in the SA state, which was at least partially caused by a coarser microstructure leading to higher levels of plasticity- and roughness-induced crack closure. In order to separate the mechanisms of crack closure and the non-closure effect of TRIP in the resistance to FCG, experiments at the load ratio R = 0.7 were conducted. Here, the threshold in the SA state was still higher than in the AR state by 1 MPa·m^0.5^. This effect was identified as crack tip shielding induced by phase transformation, an example of a non-closure shielding effect. Higher resistance to crack growth in the SA state was attributed to promoted martensitic transformation in non-favourable-oriented grain families rather than thicker martensite layers in the crack path area. The conclusions were verified by experiments at R = 0.7 and T = 150 °C, which did not reveal any notable differences in the thresholds. However, the threshold values were affected by the load-shedding gradient C = −d∆K/da, which had to be equalized in both experimental setups inside and outside the furnace. In summary, there is an intrinsic effect of microstructure on FCG rate, but crack closure still plays a major role.

### 2.4. Effect of Crack Branching

Toribio et al. [7] studied the effect of crack path branching (at the micro level) on FCG. A cracked panel subjected to tension with different symmetric and asymmetric configurations of the bifurcated crack tip was modelled using the finite element method. The crack extension occurred by cyclic blunting and re-sharpening (in agreement with the Laird–Smith mechanism), transferring material from the crack tip towards the crack flank. The bifurcated crack has a retardation effect on the growth rate in relation to a completely straight crack. Additionally, (i) if the two branches of the bifurcation have different initial projected lengths, the propagation rate is greater at the crack tip corresponding to the long-branch, and the retardation effect increases with the initial branch angle and the initial projected short-branch length, and decreases with ΔK. (ii) If the two branches of the bifurcation have identical initial projected lengths, the retardation effect increases with the initial distance between the two bifurcated crack tips, and decreases with ΔK.

### 2.5. Effect of In-Plane Constraint

A parameter of FCG is the in-plane constraint, which is usually quantified by T-stress. Galkiewicz and Janus–Galkiewicz [8] used a 2D-modified boundary layer model with cohesive elements to study the effect of T-stress on the crack behaviour. The boundary conditions corresponding to different T-stress levels caused higher FCG rates for higher levels of constraints. In fact, the lower the level of constraint was, the larger the plastic zones develop in the neighbourhood of the crack tip. Absorbing energy through plastic zones means that less energy is used to create a new surface. Additionally, the lower the level of constraint, the longer the crack remained closed, and the lower the effective SIF.

### 2.6. Spalling Analysis of Large Particles in High-Cr Steel during Thermal Fatigue

In industrial operations, work steel rolls are usually heated to 500–700 °C at the contact surface and immediately cooled with water. The cycles of heating and cooling lead to thermal fatigue. The material is exposed to constantly changing mechanical stresses and is subject to strong oxidation conditions. The process of crack formation and propagation associated with the increased spalling of large particles (over 300 µm) is not yet fully understood. Bombac et al. [9] studied the surface deterioration of high-Cr roll steel caused by the spalling of larger particles during thermal fatigue. Using a laboratory thermal fatigue test that replicates hot rolling conditions, samples were cyclically tested (up to 4500 times) at maximum cycle temperatures of 500, 600 and 700 °C, followed by water cooling. Thermal fatigue tests were interrupted after 200, 500, 1000, 2500 and 4500 thermal cycles, and the specimens were axially and radially sectioned for the analysis of microstructure and cracks. Specimens with surface deterioration were selected for analysis, revealing important influencing parameters, i.e., the combination of test temperatures, chemical composition, thermal stress and microstructural properties, leading to surface deterioration due to early spalling of larger particles.

### 2.7. Welding Joint of 6005A-T6 Aluminium Alloy

The fatigue of welds is a complex process as it involves defects, residual stresses and local changes in material and microstructure. Peng et al. [10] studied metal inert gas welding (MIG) applied to 4 mm thick 6005A-T6 aluminium alloy. All the fatigue specimens fractured at the weld toe of the lap joint, and the failure was characterized by a cleavage fracture. Crack closure induced by oxide was observed during the steady propagation of the fatigue crack. Impurities hindered crack propagation, changed its direction, and demonstrated a stepped fatigue strip distribution morphology; in the process of the main crack propagation, the initiation and propagation of small cracks were easily restricted and hindered by the main crack.

### 2.8. Fatigue Crack Initiation

In components without initial defects, a major part of the fatigue life is the initiation life. The fatigue initiation life, the initiation sites and the crack length corresponding to the transition from initiation to propagation are important issues. 

Fintová et al. [11] studied the initiation mechanisms in a AZ91 magnesium alloy tested in loading conditions ranging from low to very high cycle fatigue regimes. In low and high (below 10^7^ cycles) cycle fatigue regions, slip markings were created in the solid solution areas of the cast microstructure of the AZ91 magnesium alloy. Additionally, broken Mg_17_Al_12_ intermetallic particles were observed in the microstructure; however, the cracks almost always stopped on the particle/solid solution interface and did not propagate to the material bulk. In the very high cycle fatigue region, cracks initiated on the broken primary Mg_17_Al_12_ intermetallic particles grow towards the solid solution areas. The change in the initiation mechanism causes a fatigue limit decrease from 80 MPa, determined from low-frequency test data for 1 × 10^7^ cycles, to 60 MPa, determined from very high frequency tests for 2 × 10^9^ cycles.

Jesus et al. [12] analysed the fatigue behaviour of Ti-6Al-4V specimens manufactured by selective laser melting with internal axial channel. The effect of this inner surface was studied with a consideration of the following: hole as manufactured; hole drilled and hole threaded M4 × 0.7. The analysis of surface roughness and stress concentration effects show that: (i) The stress concentration due to internal thread caused a strong fatigue strength reduction. (ii) A good fatigue performance was obtained for internal surfaces with better finishing. (iii) The Ti-6Al-4V alloy showed a high notch sensibility. (iv) Internal surfaces produced by SLM showed significant unfused particles and a lack of fusion defects that led to a high surface roughness and lower fatigue strength, while drilling surfaces showed a better surface finish and, consequently, higher fatigue strength.

## 3. Applications

The application of knowledge to real components is the final target of fundamental research in this field. Usually, geometries and the environment are complex, and the loading patterns are irregular, making the design of components difficult.

### 3.1. Orthopaedic Implants in In Vivo Environments

Wu et al. [13] developed a literature review focused on the orthopaedic implants and prognosis of patients. Given the lack of specific understanding and consensus, previous investigations on FCG in internal fixation were relatively sparse and incomplete. This review presents a summary of recent findings on the fatigue mechanisms and fracture of implants in the initial period after surgery. The possible causes of FCG were elaborated from the perspectives of underlying crack propagation mechanisms, inappropriate use, design flaws, the elastic modulus, biocompatibility, corrosion resistance, and metal allergy. The latest technological issues and potential capabilities of implants that combine absorbable materials and shape memory alloys are discussed.

### 3.2. Diffusion-Bonded Titanium Alloy Laminates with Preset Unbonded Areas

**Figure 2:**
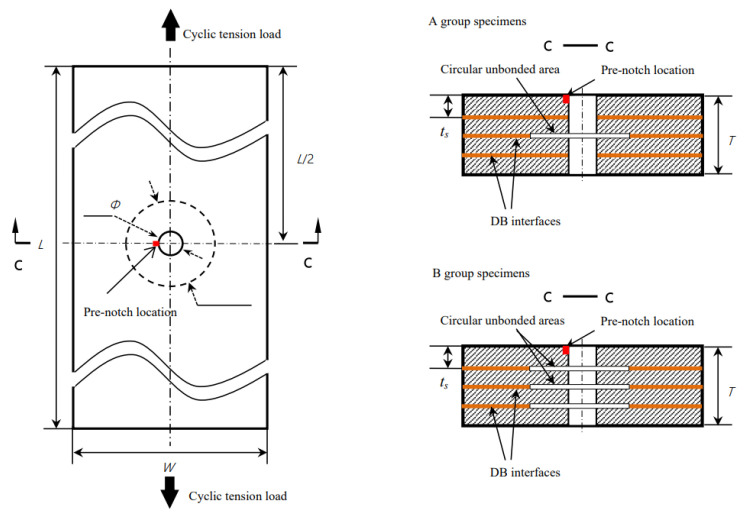
Geometry of specimens [14].

Titanium alloy laminates are manufactured by the diffusion bonding of thin sheets. The thickness and number of layers of a diffusion-bonded plate are design parameters, which can be used to adapt the material to different structural demands. The diffusion-bonded titanium alloy laminates with preset unbonded areas (DBTALPUA) have a little decline in static strength under in-plane loading, but the unbonded area can change the crack growth path and prolong its life. DBTALPUA has enormous potential in damage tolerance performance, especially when used in structures with stress concentration (e.g., structures with an open hole or joint) under in-plane loading. Liu and Liu [14] experimentally analysed eight specimens of two types of titanium laminates with unbonded areas. For the specimens that contained open holes, the unbonded areas significantly prolonged the fatigue growth life. This is because the unbonded areas can lead the crack far away from the hole edge where there is a high stress concentration. The fatigue life of Group B specimens (see Figure 2) is much longer than that of Group A. In Group B specimens, the cracks reached the unbounded areas earlier, which guided the crack away from the stress concentration zone.

### 3.3. Orthotropic Steel Decks

Orthotropic steel decks are widely used in the construction of steel bridges because of their favourable characteristics, such as large carrying capacity, low dead weight and short construction period. However, orthotropic steel decks are liable to fatigue once placed in service. Fatigue evaluation criteria based on stress amplitude are widely used, but fatigue cracks still occur. The most probable reason for this phenomenon is the underestimation of the fatigue stress caused by traffic loads.

Cheng et al. [15] discussed the validity of a traditional local approach and the multi-vehicle cooperative effect on the calculation of the fatigue stress of orthotropic decks. To fully pursue fatigue stress, the Arlequin theory, which has greater flexibility in the modelling process and stronger theoretical approval, is combined with an FE analysis. Taking a special-shaped tied arch bridge in Jiaxing City, China, as an example, full-bridge FE models, including weld details, were established with the application of Arlequin technology. The results show that the Arlequin model for deck fatigue analysis tends to be an efficient method for complete fatigue stress acquisition, whereby the vulnerable sites of orthotropic steel decks under traffic loads are defined. Vehicles near the flexible components, such as hangers or cables, can have adverse effects on the fatigue durability of decks. Additionally, the total number of vehicles and their arrangement concentration affect fatigue performance. Regardless of the gross bridge mechanics and deck deformation, the fatigue stress range is underestimated by about 30–40%, which may explain the premature cracks observed in orthotropic steel decks.

### 3.4. Rail Components

The railway industry faces rapid rail deterioration due to high axle loading, increased rollingstock speeds, and a greater service frequency to meet passenger demands. These extreme conditions lead to wear, rolling contact fatigue (RCF), and plastic deformation, causing fracture and failures if expensive rail replacement procedures are not undertaken. These damages are particularly prevalent in light rail or tram components due to the generally used lower carbon steel grades, the presence of tight radius curves in urban environments, frequent acceleration, and sudden braking. Standard maintenance procedures for light rail components use submerged arc welding techniques for repair applications. A new approach to rail maintenance uses laser cladding to deposit a metal at the rail surface in order to rebuild the worn profile or apply a coating with improved tribological properties. This is achieved using a high-energy laser that simultaneously melts a metallic powder and the rail substrate to metallurgically form a high-quality coating layer. Fasihi et al. [16] studied two cladding alloys—Stellite 6 and a newly developed martensitic stainless steel—for laser cladding on a low-carbon steel grade used for the fabrication of light rail switch blades. This study assesses the feasibility of using the laser cladding technique for light rail applications and establishes the most suitable cladding alloys and laser cladding process parameters. It was found that laser cladding with a suitable alloy was an effective technique for improving tribological properties, increasing wear resistance, and increasing the retardation of cracking in both substrates. However, the performance of the cladded components is highly dependent on the process parameters and characteristics of the deposited material.

## 4. Development of New Tools

### 4.1. Machine for VHCF in Bending

The development of new tools is of major importance for a better understanding of FCG. Mechanical structures are subject to high-frequency with small-amplitude variable loadings in aviation, aerospace, and other industrial fields. Research on the very high cycle fatigue (VHCF) regime has played an essential role over the last decade because of the development of the ultrasonic fatigue test machines. Given the limited results of existing research on bending fatigue at the VHCF regime, Yang et al. [17] developed a novel model of ultrasonic fatigue machine to test the bending of thin wall material up to VHCF. The specimen is a thin plate similar to a blade. In order to monitor the stress amplitude of the specimen and accurately record the fatigue life of the specimen in real-time, a laser-based measurement system is developed. At the bottom of the horn, the specimen was similarly fixed to a cantilever beam that was perpendicular to the vertical horn. The cyclic bending had a stress ratio of R = −1 and a frequency of 20 kHz. The specimen and the horn were fastened by a pin-hole type connection, which introduced stress concentration. An hourglass form was considered in the design process to ensure the fatigue crack occurred at the gauge section.

### 4.2. Improving Numerical Tools

Song et al. [18] proposed an adaptive mesh refinement method called ‘h-adaptive element splitting’ (h-AES) for the numerical simulation of cracks using shell elements in FEM. H-adaptivity is the splitting of finite elements in space while keeping their polynomial degree fixed. Structured coarse meshes are beneficial to the reduction in computational resources for the study of large structures. With the h-AES method, very small cracks are well-represented in these large structures without the deletion of elements. Two examples of the h-AES method for crack simulations in large structures under LEFM scenarios are presented. The numerical results were verified against analytical solutions and showed a good correspondence. The h-AES method was proven to be able to effectively reveal local details of geometry and material behaviour in numerical models, mostly consisting of structured coarse mesh.

Steel structures are widely used in engineering structures due to their high strength, light weight, and good ductility; however, under strong earthquakes, steel structures will encounter fracture damage due to ultra-low cycle fatigue (ULCF). The ULCF of structural steel occurs under cyclic large plastic strain loading, corresponding to less than 20 cycles. A simple and feasible model for predicting the ULCF life of steel structures has not yet been developed. Yu et al. [19] proposed a simplified uncoupled prediction model that considers the effect of stress triaxiality on damage and introduces a new historical-effect-related variable function, reducing the calibration work of model parameters. Specimens made of Q345qC steel, comprising circular notches with different radii, were subjected to constant-amplitude cyclic large-strain loading tests. The calibration of model parameters was made using experimental results. The main conclusions were: (1) The proposed model has fewer parameters needed for calibration when compared with the original model, and the coupling between the parameters is reduced. (2) The predicted life is very close to that of the tests. (3) Combining the incremental form of ductility consumption under monotonic loading and the nonlinearity and historical effects under cyclic loading is appropriate for calculating the ULCF damage of structural steels.

## Data Availability

Not applicable.

## References

[1] Kujawski D. (2020). Discussion and Comments on KOP and ∆K_eff_. Materials.

[2] Borges M.F., Neto D.M., Antunes F.V. (2020). Revisiting Classical Issues of Fatigue Crack Growth Using a Non-Linear Approach. Materials.

[3] Neto D.M., Borges M.F., Sérgio E.R., Antunes F.V. (2022). Effect of Residual Stresses on Fatigue Crack Growth: A Numerical Study Based on Cumulative Plastic Strain at the Crack Tip. Materials.

[4] Sérgio E.R., Antunes F.V., Borges M.F., Neto D.M. (2021). FCG Modelling Considering the Combined Effects of Cyclic Plastic Deformation and Growth of Micro-Voids. Materials.

[5] Lopez-Crespo C., Cruces A.S., Seitl S., Moreno B., Lopez-Crespo P. (2021). Estimation of the Plastic Zone in Fatigue via Micro-Indentation. Materials.

[6] Jambor M., Vojtek T., Pokorný P., Šmíd M. (2021). Effect of Solution Annealing on Fatigue Crack Propagation in the AISI 304L TRIP Steel. Materials.

[7] Toribio J., González B., Matos J.-C. (2021). Effect of the Crack Tip Bifurcation on the Plasticity-Induced Fatigue Propagation in Metallic Materials. Materials.

[8] Galkiewicz J., Janus-Galkiewicz U. (2021). The Numerical Analysis of the In-Plane Constraint Influence on the Behavior of the Crack Subjected to Cyclic Loading. Materials.

[9] Bombac D., Kugler G., Burja J., Tercelj M. (2022). Early Spalling Analysis of Large Particles in High-Cr Steel during Thermal Fatigue: Relevant Mechanisms. Materials.

[10] Peng Z., Yang S., Wang Z., Gao Z. (2022). Fatigue Property and Small Crack Propagation Mechanism of MIG Welding Joint of 6005A-T6 Aluminum Alloy. Materials.

[11] Fintová S., Trško L., Chlup Z., Pastorek F., Kajánek D., Kunz L. (2021). Fatigue Crack Initiation Change of Cast AZ91 Magnesium Alloy from Low to Very High Cycle Fatigue Region. Materials.

[12] Jesus J., Ferreira J.A.M., Borrego L., Costa J.D., Capela C. (2021). Fatigue Failure from Inner Surfaces of Additive Manufactured Ti-6Al-4V Components. Materials.

[13] Wu K., Li B., Guo J.J. (2021). Fatigue Crack Growth and Fracture of Internal Fixation Materials in In Vivo Environments—A Review. Materials.

[14] Liu Y., Liu S. (2022). Experimental Research on Fatigue Crack Growth Behavior of Diffusion-Bonded Titanium Alloy Laminates with Preset Unbonded Areas. Materials.

[15] Cheng C., Xie X., Yu W. (2021). Investigation of the Fatigue Stress of Orthotropic Steel Decks Based on an Arch Bridge with the Application of the Arlequin Method. Materials.

[16] Fasihi P., Kendall O., Abrahams R., Mutton P., Qiu C., Schläfer T., Yan W. (2022). Tribological Properties of Laser Cladded Alloys for Repair of Rail Components. Materials.

[17] Yang D., Tang S., Hu Y., Nikitin A., Wang Q., Liu Y., Li L., He C., Li Y., Xu B. (2022). A Novel Model of Ultrasonic Fatigue Test in Pure Bending. Materials.

[18] Song S., Braun M., Wiegard B., Herrnring H., Ehlers S. (2022). Combining H-Adaptivity with the Element Splitting Method for Crack Simulation in Large Structures. Materials.

[19] Yu M., Xie X., Li S. (2022). A Simplified Ductile Fracture Model for Predicting Ultra-Low Cycle Fatigue of Structural Steels. Materials.

